# Evaluation of an immunochromatographic test for the diagnosis of pulmonary tuberculosis in Madagascar

**DOI:** 10.1186/1756-0500-4-403

**Published:** 2011-10-12

**Authors:** Al-habib O Said Toihir, Vincent Richard, Vaomalala Raharimanga, Samuel H Andrianarisoa, Gabriel M Ranjalahy, Herimanana Ramarokoto, Voahangy Rasolofo

**Affiliations:** 1 Unité des Mycobactéries, Institut Pasteur, Antananarivo, Madagascar; 2 Unité d'épidémiologie, Institut Pasteur, Antananarivo, Madagascar; 3 Organisation Mondiale de la Santé (OMS), Antananarivo, Madagascar; 4 Programme National de Lutte contre la Tuberculose, Ministère de la Santé, Madagascar

## Abstract

**Background:**

To improve the diagnosis of tuberculosis (TB), more rapid diagnostic techniques such as antibody detection based on immunochromatographic methods were developed. The objective of this study was to evaluate the performance of the SD Rapid TB kit for the diagnosis of active TB with serums from patients and close contact controls in Antananarivo, Madagascar.

**Findings:**

We conducted a population-based case-control study. The sera of 60 confirmed TB patients and 60 healthy contacts paired for sex and age were tested. The controls were healthy contacts who were exposed to TB but had no clinical or radiological evidence of TB. The SD Rapid TB kit was used with serum samples according to the instructions of the manufacturer. Sensitivity, specificity, accuracy and predictive values were calculated using culture on Lövenstein-Jensen media as "gold standard".

In this study, the sensitivity of the test was 55% and the specificity 90%. The positive predictive value and the negative predictive value were 84.61% and 66.66%, respectively, for diagnosis of pulmonary TB.

**Conclusions:**

This study shows that the SD Rapid TB test is a simple and fast method. This test has a good specificity and could therefore help rule in TB if positive, but lacks adequate sensitivity.

## Background

Tuberculosis (TB) poses a significant health threat worldwide with an estimated 9 million new cases and 2 million deaths every year worldwide [1]. Morbidity associated with TB has been decreasing but Human Immunodeficiency Virus (HIV) associated TB and multidrug resistant (MDR) are on the rise [2]. According to the 2010 World Health Organization (WHO) report [1], the incidence of TB in Madagascar is estimated at 248 per 100 000 population for all forms of TB and 111/100 000 for smear-positive pulmonary TB. The prevalence of TB is estimated at 415/100 000 and the rate of human immunodeficiency virus (HIV) infection among TB patients at 0.4%.

The vast majority of TB patients live in developing countries, where the diagnosis of TB relies on the identification of acid-fast bacilli on unprocessed sputum smears using conventional light microscopy. However, 40 to 60% of patients with pulmonary disease and ~75% of patients with extrapulmonary disease are smear negative, and in this situation even contemporary culture methods take several weeks to become positive [3].

Rapid diagnostic tests for TB are needed to facilitate early treatment of TB and prevention of *Mycobacterium tuberculosis *transmission. Therefore, a number of alternative diagnostic tests that use molecular, chromatographic and immunological methods have been developed.

In this report, we evaluate an immunochromatographic test (ICT) kit, SD BioLINE Rapid TB^® ^which can detect serum antibodies directed against specific recombinant antigens of *M. tuberculosis *for the diagnosis of pulmonary TB, by determining its sensitivity and specificity as compared to standard diagnostic procedures in Antananarivo, Madagascar.

## Methods

### Patients

This study was designed as a pair matching case-control study. Between October 2007 and December 2008, 60 newly recruited sputum-positive TB patients (cases) over 15 years of age were enrolled at the principal anti-TB center of Antananarivo, the capital of Madagascar, where the annual incidence of newly diagnosed sputum smear-positive patients is about 80 cases per 100, 000 inhabitants according to the National TB Control Programme (NTCP) [4]. The patients were treated according to the NTCP strategy and were followed up by the clinical physicians.

The 60 subjects selected for the patients group had a median age of 35.5 years (age range, 15-59 years) and 54 (45%) were males, 66 (55%) were females.

### Control group

The control group consisted of 60 individuals selected randomly from apparently healthy household or family contacts of TB patients, and matched for sex and age with case subjects (difference between birthdays less than 5 years). All members of control group had no previous history of TB, no signs or symptoms suggestive of pulmonary TB, no evidence of TB on chest X rays. The household contacts (HCs) of the included index cases were visited at home by the study physicians and were invited to join the study. They were included if they were ≥15 year old and had been living in the same house as the case patient for at least 6 months.

Household contacts controls underwent a PPD skin test (10 units; tuberculin purified protein derivative; Aventis Pasteur). Induration was recorded after 72 h.

All subjects selected for the control group were HIV and PPD skin test negative.

Serum samples were obtained from almost all patients before initiation of antituberculosis treatment and stored at -20°C until tested. All patients and controls participated in the study were vaccinated with *Mycobacterium bovis *BCG. Individuals with a diagnosis of TB disease were referred to the antituberculosis center for treatment.

The study was approved by the National Ethics Committee to the Ministry of Health in Madagascar.

### Samples collection

All TB cases were screened for TB independently by microscopy, culture and SD Rapid TB to evaluate the performance of the test under field conditions. A questionnaire was filled for each patient with basic clinical and demographic information after taking written consent. Three sputum samples were obtained from each of the 60 patients using routine techniques, and the samples were processed in the Chest Clinic according to established norms [5]. Venous blood was drawn from the study participants and placed into one 5 ml dry vacutainer tube without preservative or anticoagulant for separation of serum. Each serum sample was divided into two parts: part was used for HIV testing and the other was frozen at -20°C for SD Rapid TB testing.

### SD Rapid TB interpretation

**Figure 1:**
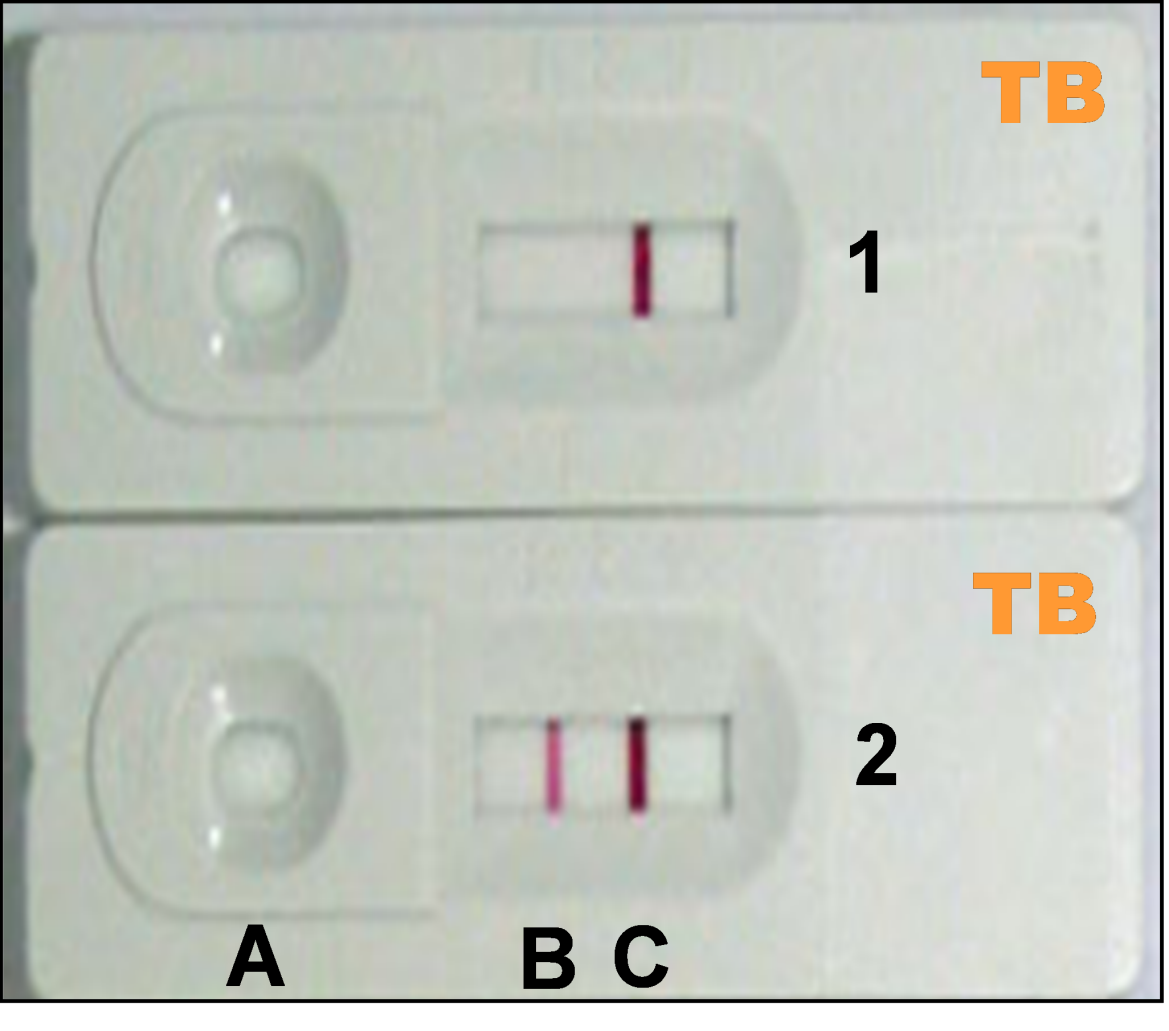
**Interpretation of SD Rapid TB results**. (A) Sample insertion. (B) Test result. (C) Internal control; 1. Negative result; 2. Positive result. When serum or plasma applied (A), it flows past the antigen line (B). Bound antibody is detected by a goat anti-human IgG antibody conjugated to colloidal gold particles which produces one pink line when bound to human antibody. The test is considered positive when at least the antigen line and the control line (C) develop a pink color.

The SD Rapid TB test (Standard Diagnostic, Inc; Cat. No. 08FK10) is a chromatographic immunoassay for qualitative detection of antibodies to *M. tuberculosis *in human serum. Briefly, the test consists of a cardboard folding device containing a nitrocellulose strip and absorbent pads. Antigen secreted by *M. tuberculosis *during active infection is immobilized on a line across the strip. When serum (100 μl) is applied, it flows past the antigen line. If specific antibody to the antigen is present, it binds to the line. Bound antibody is detected by a goat anti-human immunoglobulin G antibody conjugated to colloidal gold particles which give a pink band when bound to human antibody. Test card has one control line to indicate the validity of the test procedure and its working condition. Control and test lines appeared within 15 minutes in a reading window. SD Rapid TB was performed and the results were interpreted according to the instructions of the manufacturer by two independent readers who were blind to the clinical status of the subjects and to the TB diagnosis results. The presence of two bands (control and test line) indicates a positive result (Figure 1). Each SD Rapid TB was saved as documentation for future reference. Only tests from one batch were used (Manufacture June 07, expiry January 09 batch no. 046022).

### Clinical and laboratory evaluation

Two spot and one overnight sputum specimens were collected from each patient.

The sputa were decontaminated by the sodium dodecyl sulfate method of Tacquet and Tison [5]. Fluorescence microscopy was performed for the direct detection of mycobacteria by the auramine staining method [6]. One drop of the decontaminated sputum was fixed on a slide, stained with auramine-phenol, and examined under a fluorescence microscope (objective, ×40) for acid-fast bacilli (AFB). Sputum smear microscopy was considered positive when AFB were seen in at least two out of the three specimens. The remaining decontaminated specimen was inoculated into two tubes of standard LJ medium (Diagnostics Pasteur, Paris, France) and on one tube of LJ medium without glycerol but supplemented with 0.5% pyruvate. Culture said to be positive when at least one colony of *M. tuberculosis *was grown, and negative if there was no growth after 8 week. The numbers of colony forming unit (CFU) growing in each LJ tube were counted. Mycobacterial isolates were identified according to growth on LJ medium, colony morphology, and biochemical tests for the following: niacin production, catalase, urease, and nitrate reductase [6]. All subjects included were serotested for HIV.

### Data analysis

The performance of SD Rapid TB was expressed by calculating the sensitivity, specificity, positive predictive values (PPV) and negative predictive values (NPV) for TB separately taking culture results as gold standard (According to Grange and Laszlo 1990 [7]). Data were double entered, validated and analysed using Epi Info™ 3.3.6 software (CDC Atlanta GA, USA). Proportions were compared using the chi-square test.

## Results

The serum samples included in the study were from patients in whom the disease had been confirmed by isolation of the tubercle bacillus on culture (LJ). Sera were also collected from 263 contacts of TB patients. After matching for sex and age, 120 individuals were selected and enrolled (60 cases/60 age and sex-matched control subjects). All subjects were Malagasy and HIV negative. None of the control subjects became ill during the 1 year follow-up. No samples gave indeterminate results with the SD Rapid TB test. Of the 60 AFB smear-positive and culture-positive patients evaluated, 33 (55%, 95%CI: [0.425 to 0.669]) were positive with the SD Rapid TB test. Positive results were also obtained for six (10%) of the 60 apparently healthy contacts, so the specificity of the test was 90%, 95%CI: [0.799 to 0.953]. There was a statistically significant difference between the results obtained with the SD Rapid TB test and culture (McNemar test, *P *< 0.0001). The difference between ICT results in pulmonary TB patients and control subjects was statistically significant (P < 0.0001). The PPV and NPV of the SD Rapid TB were 84.62% and 66.67% respectively.

## Discussion

TB is a major public health problem throughout the world, particularly in developing country. Lack of sensitivity in smear examination, non-specificity of radiological findings, and extended turn around time of *M. tuberculosis *culture have necessitated exploring the utility of serodiagnostic of TB. TB serology based on antibody and antigen detection for diagnosing and monitoring tubercular infection with low cost and flexibility to adapt to small laboratories may be a tremendous advantage to the developing countries. A number of serologic tests were developed for detection of antibody against to *M. tuberculosis*, but none of them has found widespread clinical use [8].

This study was designed to evaluate the diagnostic potential of SD BioLINE Rapid TB^® ^test for the detection of TB. We found that the specificity (90%) was similar to the results for adult ICT-TB reported by other authors (between 88% and 95%) [8,9]. We also found that the sensitivity was low (55%). Similarly performance has previously been reported for other serological tests to identify pulmonary TB patients, in particular, sensitivity is poor to moderate (16% to 57%) for seven serological tests, including two immunochromatographic tests (ICT Tuberculosis and RAPID TEST TB) and five enzyme-linked immunosorbent assays (TUBERCULOSIS IgA enzyme immunoassay, PATHOZYME-TB complex, PATHOZYME-MYCO IgG, PATHOZYME-MYCO IgA, and PATHOZYME-MYCO IgM) [10].

Discrepancies between studies in the results of a commercially available serological test can most often be attributed to differences in the populations studied.

The performance of the rapid test kit as concerns sensitivity, by comparison with direct sputum smear microscopy and culture, was unsatisfactory. The low sensitivity of this assay seems to be because the antibody response to a particular antigen may not be universal, as established by Lyashchenko et al. [11].

The first antigen used in this assay is a highly specific recombinant 38 kDa antigen from *M. tuberculosis *which has been produced and purified from *Escherichia coli*[12]. This antigen has been reported to be the single most important antigen for the serodiagnosis of TB [13]. The 16-kDa antigen (second antigen) is reported to be a prominent antigen of *M. tuberculosis*, with epitopes restricted to *M. tuberculosis *complex based on B-cell recognition [14]. ESAT-6 was characterized and purified in 1995 by Sorensen et al. [15], and has since been used in various studies as an antigen with diagnostic potential: It was found to be specific for *M. tuberculosis *and absent from *M. bovis *BCG and most environmental mycobacteria.

This new test detects IgA antibodies in serum. An increase in circulating IgA has been found in TB patients, but also in sera from both patients with other infectious respiratory diseases and patients with rheumatic and auto-immune diseases and persistent infections [16-19] Thus, the detection of IgA may increase the sensitivity of the detection of pulmonary TB, but it may also be the cause of the false-positive results obtained in the group of non-TB patients.

Diagnostic tests provide information that guides health care providers' decisions about initiating therapy. However, the efficacy of diagnostic tests depends on characteristics of the population. The prevalence of a disease is a well-known and powerful determinant of how useful for the clinician a diagnostic test for the disease will be. Its consequences for diagnostic test performance (*e.g*. the probability of a positive/negative result) are taken into account through the positive and negative predictive values. Indeed, the diagnostic value of a given test in clinical practice depends on its positive and negative predictive values, and these values vary markedly with the prevalence of the disease in the relevant community [20]. We calculated PPVs and NPVs using in several hypothetical pre-test probability estimates. The utility of the SD Rapid TB kit as a diagnostic test in a group of patients with TB, with pre-test probability of TB hypothesized from 10% to 100% showed that the cut-off value for the SD Rapid TB kit for best sensitivity and specificity was in the range of TB prevalence between 35% and 45%. However, our study also had limitations on the estimation of diagnostic accuracy generated by using cases and controls study [21]. Often, mild cases that are difficult to diagnose are omitted from case-control studies, causing an overestimation of sensitivity as well as specificity.

This study, although limited by the small number of volunteers, highlights the validity of the new ICT for detection of TB in endemic area.

The sensitivity is low as compared to other studies due to the reason that some people show poor antibody production due to genetic variations and differences in study populations [22]. One of the major problems in the serodiagnosis of TB has been heterologous antibody responses to various *M. tuberculosis *antigens. It seems, therefore, that more than one antigen is required for increased sensitivity. This approach may result in a lower specificity despite the use of *M. tuberculosis *specific antigens. Thus, performance of this ICT can be improved by using more purified specific antigens and the usefulness of the test should be investigated greater in endemic areas. Data obtained in this study on the quality of antibody response in the ICT positive samples, might be used to improve the performance of the test.

## Conclusions

In conclusion, the test cannot be used as a replacement for smear microscopy for the diagnosis of pulmonary TB because of its poor sensitivity. However, it may be worth assessing this kit, in future investigations, for the detection of extrapulmonary TB or pediatric TB, that is situation in which bacteriologic confirmation is often difficult [23,24]. Otherwise, the SD BioLINE Rapid TB^® ^kit, with its acceptable specificity, and despite inadequate sensitivity, could help rule in TB if positive, especially in endemic countries where the prevalence is between 35% and 45%. A better understanding of antibody responses in TB patients may facilitate the development of more sensitive and specific antibody-based methods for the TB diagnosis.

## Competing interests

The authors declare that they have no competing interests.

## Authors' contributions

AOST conceived and designed the study, conducted the laboratory studies and drafted the manuscript. VR participated in the conception and design of the study, and performed the statistical analyses. VR participated in the study design and acquisition of data. SHA, GMR and HR participated in the study design. VR participated in the conception and design of the study, and helped in writing of the manuscript. All authors read and approved the final version of the manuscript.
